# Chronic Inflammation-Related HPV: A Driving Force Speeds Oropharyngeal Carcinogenesis

**DOI:** 10.1371/journal.pone.0133681

**Published:** 2015-07-20

**Authors:** Xin Liu, Xiangrui Ma, Zhengge Lei, Hao Feng, Shasha Wang, Xiao Cen, Shiyu Gao, Yaping Jiang, Jian Jiang, Qianming Chen, Yajie Tang, Yaling Tang, Xinhua Liang

**Affiliations:** 1 State Key Laboratory of Oral Diseases, West China Hospital of Stomatology (Sichuan University), Chengdu, China; 2 Key Laboratory of Fermentation Engineering (Ministry of Education), Hubei University of Technology, Wuhan, China; 3 Department of Oral Pathology, West China Hospital of Stomatology (Sichuan University), Chengdu, China; 4 Department of Oral and Maxillofacial Surgery, West China Hospital of Stomatology (Sichuan University), Chengdu, China; Georgetown University, UNITED STATES

## Abstract

Oropharyngeal squamous cell carcinoma (OPSCC) has been known to be a highly aggressive disease associated with human papilloma virus (HPV) infection. To investigate the relationship between HPV and chronic inflammation in oropharyngeal carcinogenesis, we collected 140 oral mucous fresh specimens including 50 OPSCC patients, 50 cancer in situ, 30 precancerous lesions, and 10 normal oral mucous. Our data demonstrated that there was a significantly higher proportion of severe chronic inflammation in dysplastic epithelia in comparison with that in normal tissues (*P*<0.001). The positive rate of HPV 16 was parallel with the chronic inflammation degrees from mild to severe inflammation (*P*<0.05). The positive rate of HPV 16 was progressively improved with the malignant progression of oral mucous (*P*<0.05). In addition, CD11b+ LIN- HLA-DR-CD33+ MDSCs were a critical cell population that mediates inflammation response and immune suppression in HPV-positive OPSCC. These indicated that persistent chronic inflammation-related HPV infection might drive oropharyngeal carcinogenesis and MDSCs might pay an important role during this process. Thus, a combination of HPV infection and inflammation expression might become a helpful biomedical marker to predict oropharyngeal carcinogenesis.

## Introduction

Head and neck squamous cell carcinoma (HNSCC) includes cancers arising from the lining epithelium of the oral cavity, larynx, pharynx, and nasopharynx. High-risk human papilloma virus (HPV) has been recognized as an important risk factor in a subset of oropharyngeal squamous cell carcinomas (OPSCC) and HPV-positive OPSCC has a more favorable prognosis compared to HPV-negative OPSCC[1–4]. The incidence of HPV-positive OPSCC in the United States enhanced at approximately 7.5% per year while the incidence of HPV-negative reduced in parallel with smoking, so the percentage of HPV-positive OPSCC enhanced from less than 20% to more than 70% from 1988 to 2004[5]. However, only a small part of HPV infection evolve to cancer whereas the vast majority of HPV infection lead to no or only mild abnormalities. Ang et al. [6] showed that the death risk of HPV-positive OPSCC significantly improved with each additional pack-year of tobacco smoking, indicating that HPV state is not a sufficient etiology of carcinogenesis and demands the accumulation of additional factors.

Chronic inflammation is a well-documented risk factor for carcinogenesis. The link between cancer and inflammation was first discovered by Rudolf Virchow nearly 150 years ago, on the basis of observations that a large number of leukocytes infiltrated in tumor tissues [7]. Later on, the association of ulcerative colitis, chronic gastritis, hepatitis and chronic pancreatitis with colon, gastric, liver and pancreatic carcinomas has been uncovered [8]. Recently, epidemiologic and clinical studies demonstrated that about 15%-20% of all human solid tumors may result from chronic inflammation [9, 10]. While the influence of inflammation on tumor initiation and progression is well revealed [9], the association of inflammation with HPV infections in carcinogenesis is much less understood. The majority of present reports on inflammation and HPV-positive OPSCC mainly focused on the relationship between periodontitis and HPV-positive OPSCC. Tezal et al [11] reported that periodontitis may be associated with HPV status of HNSCC. Han et al [12] showed that chronic periodontitis may facilitate the acquisition and persistence of oral HPV infection, a recently emerging risk factor for HNSCC. Thus, the role of inflammation and HPV infection in OPSCC tumorigenesis is needed to be clarified.

In this present study, we investigated the relationship between HPV status and chronic inflammation in normal oral mucous, dysplasia, as well as cancer in situ and OPSCC. The results demonstrated that HPV status in normal and non- normal tissues correlated with enhanced chronic inflammation and was also associated with progression of precancerous lesions, suggesting a pivotal role of chronic inflammation in HPV-positive oropharyngeal carcinogenesis.

## Material and Methods

### Ethics statement

This study was approved by the Institutional Ethics Committee of the West China College of Stomatology, Sichuan University, China.

### Patients and sample collection

A total of 140 tissue samples and blood including 50 OPSCC patients, 50 cancer in situ, 30 precancerous lesions, and 10 normal oral mucous (from the benign oral tumor patients) were obtained from the Department of Oral and Maxillofacial Surgery, West China Hospital of Stomatology, Sichuan University between 2012 and 2015 (Patients who had received preoperative chemotherapy, hormone therapy or radiotherapy were excluded). Part of fresh samples with cancer, cancer in situ, precancerous lesions, and normal oral mucous were fixed, dehydrated and paraffin embedded, and their pathological features and diagnoses were verified by two pathologists with hematoxylin and eosin staining. Part of fresh samples and blood of cancer, cancer in situ, precancerous lesions, and normal oral mucous was examined by flow cytometry. Every patient signed separate informed consent forms for sampling and molecular analysis. This study was approved by the Institutional Ethics Committee of the West China College of Stomatology, Sichuan University, China.

### Evaluation of oral mucous inflammation

Oral mucous inflammation was assessed by counting the number of neutrophils and lymphocytes observed in microscope fields on Pap slides from each study subject. Slides were observed at × 1000 to identify neutrophils and lymphocytes by their nuclei morphology. The numbers of neutrophils and lymphocytes were counted in five nonadjacent fields, which were not contaminated by squamous epithelial cells. Importantly, to minimize bias, readings and countings of Pap slides for cytopathology were independently done (Tang YL and Jiang YP).

### HPV infection status

Frozen biopsies were cut in half for parallel RNA and genomic DNA extraction with the TRIzol reagent (Invitrogen) and the QIAamp Mini Kit (Qiagen), respectively. For PCR amplification of the HPV 16, 100 ng of DNA was amplified using consensus primers HPV 16 (457-bp product). All samples were run with a negative (H_2_O) and positive control (DNA from HeLa cell line). The integrity of the PCR products was visualized by agarose gel electrophoresis. Samples found to be positive for HPV by PCR were confirmed using p16 staining by immunohistochemistry (IHC). PCR primer sequence for HPV 16 is Forward 5- CAC AGT TAT GCA CAG AGC TGC-3; Reverse 5-CATATATTCATGCAATGTAGGTGTA-3[13].

### Quantitative real-time reverse transcriptase-PCR

The total RNA of specimens was collected with TRIzol reagent (Invitrogen) and treated with RNase-free DNase I (Takara) to avoid genomic DNA contamination. The complementary (c)DNA was stored at −80°C until used for PCR. PCR amplification of the cDNA template was done using Thunderbird SYBR qPCR mix (TOYOBO) on ABI PRISM 7300 sequence detection system (Applied Biosystems). The primers of IFN-γ, IL-10, IL-12 and IL-2 were used as the previous publication[14].

### Immuohistochemistory (IHC)

Paraffin-embedded samples were serially sectioned at 4 μm and IHC of P16 and MPO was performed on 4-mm-cut representative sections by the streptavidin- peroxidase method followed as previously described [15]. PBS was substituted for the primary antibody as negative controls.

### Flow cytometry

Tissues were trim into 1-2mm^3^ tissue block and put into burnisher. Then cell suspension was collected and centrifugated. To blood specimen, heparin sodium and PBS/Hanks was added into blood of individuals. Ficol was used to separate lymphocyte. Serum free medium containing 1% BSA was added into cell suspension, and then incubated on ice for 10 minutes. PE-Cy Mouse Anti-Human CD11b, FITC- Mouse Anti-Human LIN, APC Mouse Anti-Human HLA-DR, PE Mouse Anti-Human CD33 (BD Biosciences) were added to cell suspension and incubated on ice for 30 minutes. Cells were washed and resuspended in 500 mL buffer and analyzed using Flow Cytometer (Cytomic FC500, Beckman).

### Statistical analysis

All statistical analyses were performed using SPSS 13.0 (SPSS Inc., Chicago, IL USA). One-way analysis of variance was used for labeling index analysis in multiple groups. The difference of labeling index between two groups was evaluated with t-test. *P*<0.05 was considered statistically significant.

## Results

### Chronic inflammation correlates with the progression of oropharyngeal carcinogenesis

**Fig 1:**
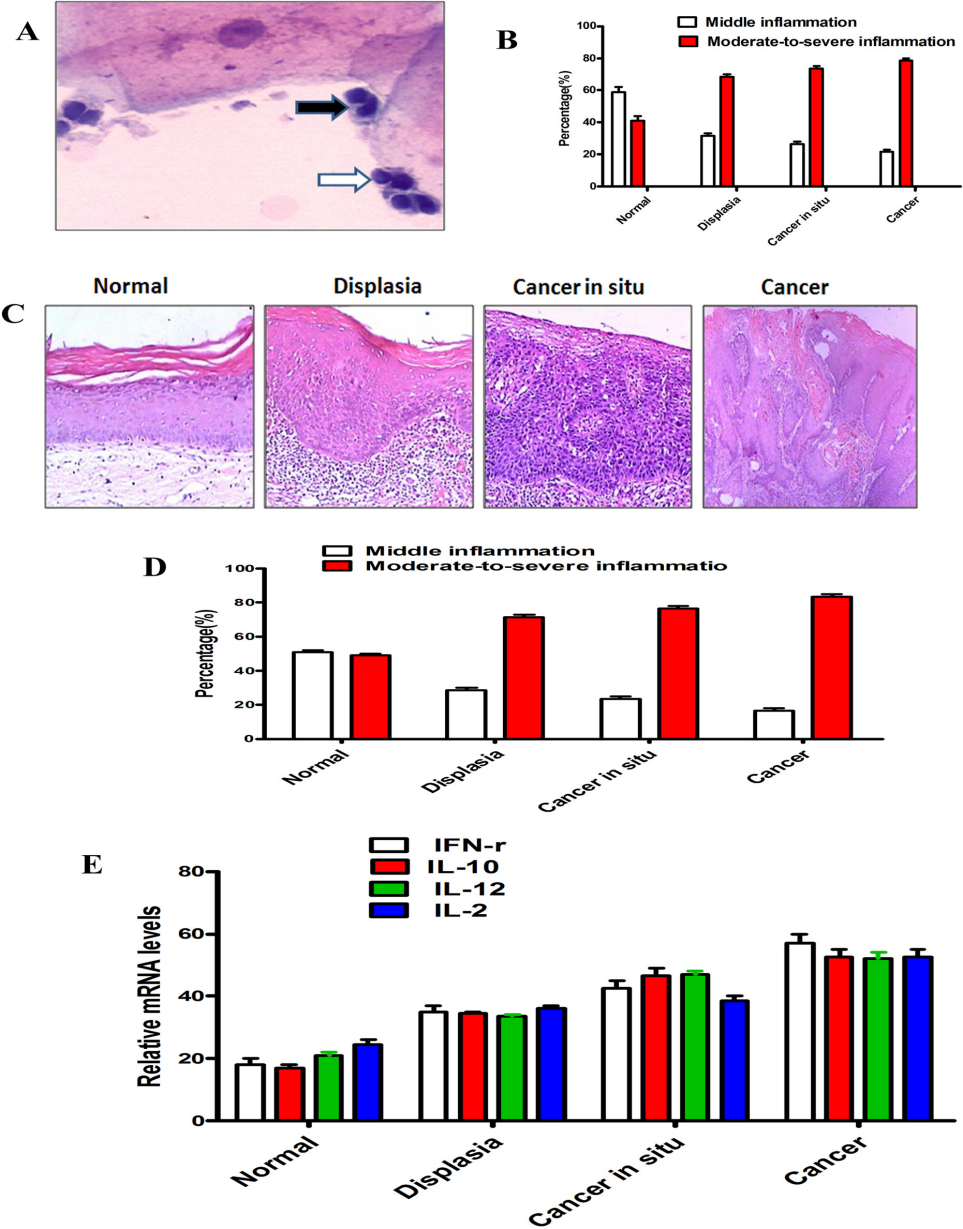
Chronic inflammation correlates with the progression of oropharyngeal carcinogenesis. (A) The morphology of neutrophils (Black arrow) and lymphocytes (White arrow) in cell smear. (B) Percentage of different degrees of chronic inflammation in cell smear of indicated types of tissues were shown. The results showed that there was significantly difference between the percentage of moderate-severe inflammation in the cell smear of dysplasia and normal oral mucous (*P*<0.001), and the inflammation degrees showed no different in the cell smear of dysplasia, cancer in situ and cancer (*P*>0.05). Error bars represent the mean±SD of triplicate experiments. (C) Representative image of normal oral mucosa, dysplasia, cancer in situ and cancer with mild inflammation. (D) Percentage of different degrees of chronic inflammation in indicated types of tissues are shown. The results showed that there was significantly difference between the percentage of moderate-severe inflammation in the tissues of dysplasia and normal oral mucous (*P*<0.001), and the inflammation degrees showed no different in dysplasia, cancer in situ and cancer specimens (*P*>0.05). Error bars represent the mean±SD of triplicate experiments. (E) Transcription levels of IFN-γ, IL-10, IL-12, and IL-2 in normal oral mucosa, dysplasia, cancer in situ and cancer tissue, relative to GAPDH, determined by quantitative RT-PCR. Error bars represent the mean±SD of triplicate experiments.

To clarify the possible role of chronic inflammation in oropharyngeal carcinogenesis, we observed the morphology of neutrophils and lymphocytes and counted their number in microscope fields on smear slides from 140 samples including the specimens of 10 normal oral mucous, 30 dysplasia, 50 caner in situ and 50 oropharyngeal cancer (Fig 1A). The number of neutrophils and lymphocytes was averaged and scored as mild inflammation (0–5 neutrophils and lymphocytes/field), moderate inflammation (6–30 neutrophils and lymphocytes/field), and severe inflammation (>30 neutrophils and lymphocytes/field). The results showed that the percentage of moderate-severe inflammation in the specimens of dysplasia, cancer in situ and cancer was much higher than that in normal oral mucous (*P*<0.001, Fig 1B), and the inflammation degrees were no significantly different in dysplasia, cancer in situ and cancer specimens (*P*>0.05, Fig 1B), indicating that there might be some relationship between chronic inflammation and OPSCC carcinogenesis.

Then, to confirm the results of the cytological examination in oral mucous smear, we analyzed inflammation status in tissues with different pathological types in microscope fields. The results showed that severe chronic inflammation was more frequently observed in dysplastic tissues (75.90%), such as lichen planus and leukoplakia, compared to normal oral mucous (48.20%, *P*<0.001, Fig 1C and 1D), indicating chronic inflammation involved in OPSCC carcinogenesis. The process of inflammation has been shown to consist of a complex cascade of events involving the participation of cytokines and chemokines, as well as the inflammation cells that produce them, which act collectively in the activation and regulation of the inflammation response [16–20]. Herein, the mRNA expression of cytokines including IFN-γ, IL-10, IL-12, and IL-2 was measured in samples by RT-PCR to reveal the levels of inflammation cytokines. As Fig 1E was shown, the mRNA levels of cytokine IFN-γ, IL-10, IL-12, and IL-2 has been elevated in dysplasia, caner in situ and oropharyngeal cancer group, compared to oral mucous specimens. All the results confirmed a potential link between chronic inflammation and OPSCC carcinogenesis.

### HPV infection was parallel with the severity of chronic inflammation

**Fig 2:**
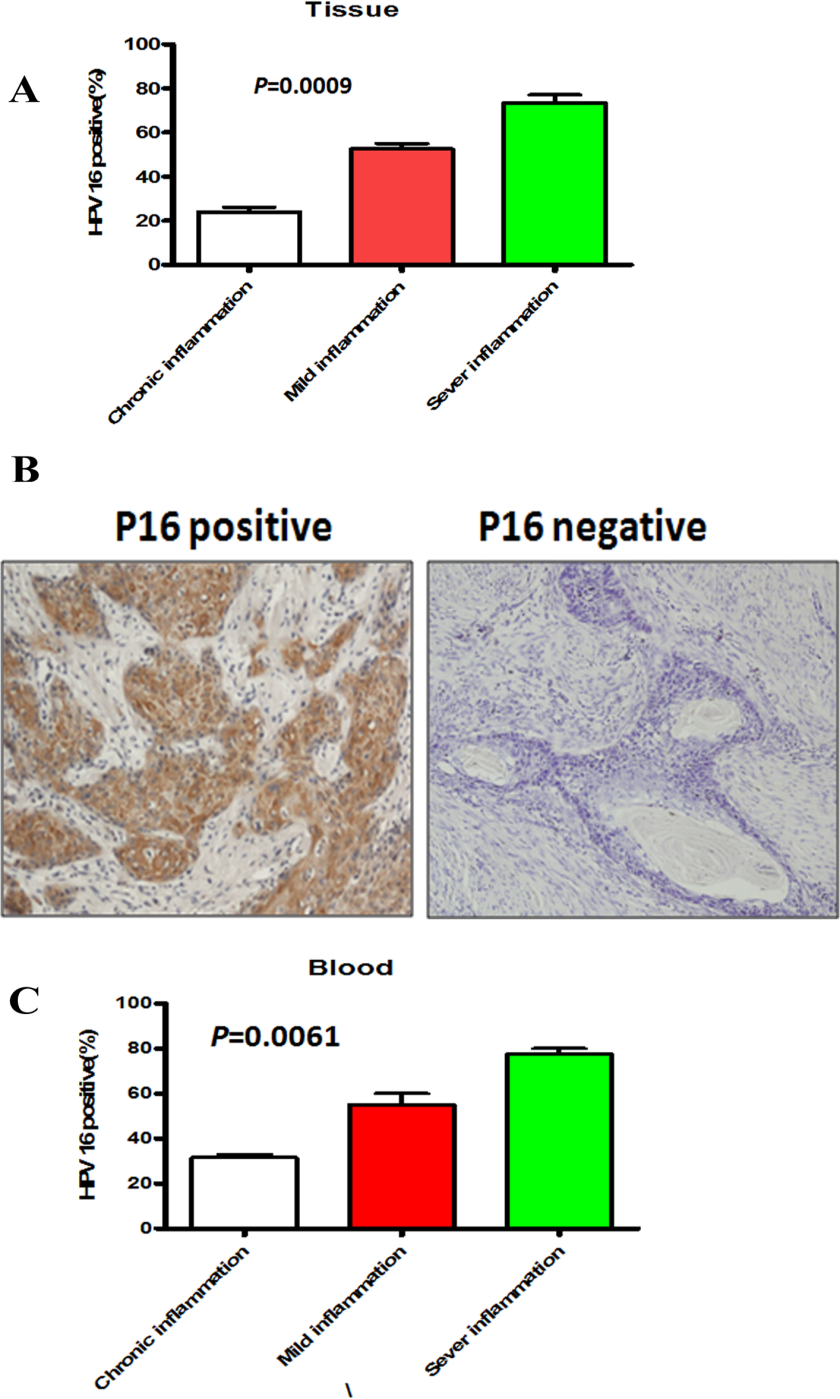
HPV infection correlates with chronic inflammation. (A) Percentage of HPV 16 positive infection in patient tissues with different degrees of chronic inflammation was shown. Error bars represent the mean±SD of triplicate experiments. (B) Representative immunohistochemical image of HPV 16 in cancer tissues with mild inflammation. (C) Percentage of HPV 16 positive infection in patient blood with different degrees of chronic inflammation was shown. Error bars represent the mean±SD of triplicate experiments.

Laboratorial and epidemiological investigations have strongly implicated HPV infection as the cause of OPSCC. However, only few HPV infection progress to OPSCC. Thus, based on the close relationship between chronic inflammation and OPSCC carcinogenesis, we hypothesized that chronic inflammation serves as the intermediate to bridge the gap between HPV infection and oropharyngeal carcinogenesis. We attempted to investigate the actual status of HPV in the above tissues with various degrees of chronic inflammation, divided into mild, moderate, and severe inflammation group. HPV DNA testing analysis revealed a significant increase rate of HPV 16 positive in tissues with severe chronic inflammation compared to samples with mild and moderate inflammation (*P* = 0.0009, Fig 2A). The same results were obtained by the IHC staining of P16 in these tissues (Fig 2B). To further ascertain this data, we subsequently sought to measure HPV 16 DNA in patient bloods by PCR. Consistent with the tissue results of HPV infection, HPV 16 was also detected with stronger signal in the sever degree of chronic inflammation compared with mild inflammation tissues (*P* = 0.0061, Fig 2C). These results suggested that chronic inflammation, to some extent, was associated with HPV 16 infection in oral tissues. In accord with our hypothesis, chronic inflammation might contribute to HPV infection to progress to oropharyngeal carcinogenesis.

### Inflammation-related HPV correlated with OPSCC progression

**Fig 3:**
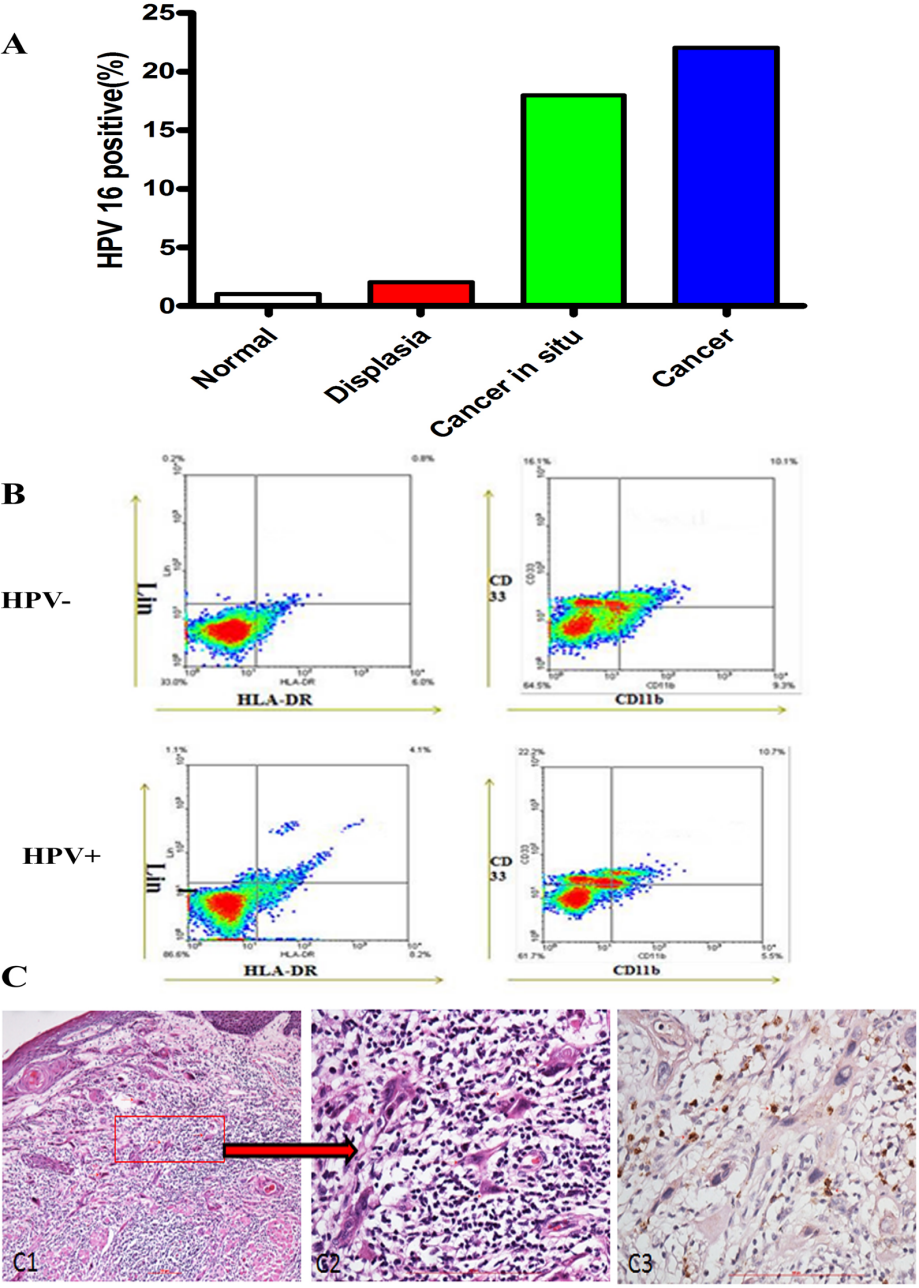
HPV infection correlates with the progression of oropharyngeal carcinogenesis. (A) Percentage of HPV 16 positive infection in patient tissues with normal oral mucosa, dysplasia, cancer in situ and cancer was shown. (B) Representative flow cytometry image of CD11b+ LIN- HLA-DR- CD33+ MDSCs in tissues of OPSCC patients with HPV-negative and HPV-positive. We first examined the percentage of LIN- HLA-DR- cells, and then screened the percentage of CD11b+ CD33+ cells in LIN- HLA-DR- cells. This image showed that the percentage of CD11b+ LIN- HLA-DR- CD33+ MDSCs in HPV-negative and HPV-positive OPSCC patients was 9.39% and 13.81%, respectively. (C) Representative immunohistochemical image (C3) of MPO in cancer tissues of OPSCC patients. C1 and C2 were H& E staining and C2 was amplification of C1.

We have now showed the close relationship between HPV infection and chronic inflammation, as well as the relevance of inflammation and oropharyngeal carcinogenesis. Then we hypothesized that HPV infection is supposed to be evident in serous inflammation precancerous lesions and cancer tissue. To test our hypothesis, we statistically analyzed the association of HPV 16 infection with normal oral mucous specimens, dysplasia, caner in situ and oropharyngeal cancer. The result showed that the positive rate of HPV 16 progressively increased in the sequential stages from histology-normal epithelia to cancer (*P*<0.05, Fig 3A), indicating that HPV infection in oral mucous may be the source of its carcinogenesis. Further, the association between HPV 16, inflammation, and cancer demonstrated by the Chi-square test showed that there was significantly co-relationship between HPV 16 infection and inflammation in cancer(*P* <0.05).

### Overexpression of MDSCs in HPV-positive OPSCC patients

Myeloid-derived suppressor cells (MDSCs), previously called immature myeloid cells [21] or myeloid suppressor cells[22–24], is a major contributor in facilitating tumor-induced immune suppression, induced by a variety of factors including vascular endothelial growth factor [11], GM-CSF[25, 26], and pro-inflammation cytokines such as IL-1 [27, 28]. Here, we examined the number of MDSCs in HPV 16 infection with normal oral mucous specimens, dysplasia, caner in situ and oropharyngeal cancer using the PE-Cy mouse anti-human CD11b, FITC- mouse anti-human LIN, APC mouse anti-human HLA-DR, PE mouse anti-human CD33. Flow cytometry results showed that CD11b+ LIN- HLA-DR- CD33+ MDSCs was obviously up-regulated in the specimens of HPV 16 infection with dysplasia, caner in situ and oropharyngeal cancer in comparsion with normal oral mucous(Fig 3B). The same results were obtained in the blood of cases. Moreover, we used MPO, the marker of MDSCs in clinic cases, to examine the expression of the specimens of MDSCs in oral mucous by immuohistochemistory (Fig 3C). The result showed that MPO was higher expressed in HPV-positive OPSCC patients than normal oral mucous(*P* <0.05). These data confirmed that MDSCs are a critical cell population that mediates inflammation response and immune suppression in HPV-positive OPSCC patients.

### Combination of the expression of HPV infection and chronic inflammation to forecast oropharyngeal carcinogenesis

To investigate the accumulative effects of HPV-positive, HPV-negative, and mild inflammation, severe inflammation expression on the OPSCC progression, we divided these 50 OPSCC patients into three groups according to the number of positive markers from HPV and inflammation. The patients were scored according to the number of positive markers: 1 (one positive), 2 (two positive) and 3 (no expression of the two markers). The results of regression analysis showed that patients with over expression of HPV and inflammation (score 2) had a highest risk to suffer from OPSCC (*P* = 0.001). This indicated that HPV infection combined with inflammation might become a helpful biomedical marker for forecasting malignant transformation of oropharyngeal mucous.

## Discussion

HNSCC has continued to have poor prognosis despite the improvement of treatment with surgery, chemotherapy and radiation therapy [29]. However, HPV-positive OPSCC has better prognosis than HPV-negative OPSCC, which pushed us to search the possible clues of tumorigenesis and malignant progression in OPSCC patients. De Cecco et al have classified HNSCC into the subtypes designed as immunoreactive, inflammatory, HPV-like, classical, hypoxia associated, and mesenchymal, based on their main biological characteristics and de-regulated signaling pathways [30]. This study evaluated the relationship between the two subtypes (inflammatory and HPV-like) in the specimens of normal oral mucous, dysplasia, cancer in situ and cancer in order to provide pathological evidence in human oral mucous tissues for the critical role of chronic inflammation-related HPV infection in oropharyngeal carcinogenesis.

Carcinogenesis including OPSCC is a multi-step process involving progression from precancerous cells to cancer cells that undergo a succession of intermediate stages in order to uncontrollably proliferate, metastasize, and reach a fully malignant functional phenotype [31]. During this process, inflammation cells and cytokine, ranging in distribution and composition might be recruited to the precancerous areas to produce a variety of cytotoxic mediators [9]. This study first demonstrates significantly different inflammation degrees in patients with normal oral mucous, dysplasia, cancer in situ and OPSCC, pointing out that inflammation may be an initiating factor of oropharyngeal carcinogenesis. Oral physiological and pathological bacterium may provide the evident of existence of chronic inflammation. Previous studies revealed that certain malignancies arise from sites affected by severe chronic inflammation [32]. Farhan-Alanie et al [33] reported that the activated systemic inflammation seems to be a powerful adverse prognostic indicator in resectable oral squamous cell carcinoma. Woodford et al [34] showed that HNSCC tissues produced increased levels of TGF-β compared to premalignant lesions, and skewed normal spleen cells toward the Treg phenotype. Singh et al [35] showed that TNF-α (-238) G/A polymorphism was significantly associated with oral squamous cell carcinoma, however, TNF-α (-308) G/A polymorphism was not associated with oral squamous cell carcinoma. These data supported that inflammation could initiate or accelerate malignant transformation in the affected oral mucous as a driving force.

During many microorganisms studied, viral infections are suggested to involve in cancer development especially that was caused by HPV, which combined various inflammation cells and cytokines in tumor microenvironment to contribute to carcinogenesis, and tumors have been called as “wounds that do not heal” [36]. The increasing studies showed that inflammation may be an important co-factor, contributing to the progression of cervical cancer with HPV in past decades [37–39]. However, empirical evidence of this same concept in OPSCC is lacking. In this study, we investigated the relationship between HPV infection state and chronic inflammation. Our results revealed that HPV 16 DNA in tissues and blood of specimens was detected with stronger signal in severe inflammation compared with mild inflammation, suggesting a close relationship between HPV infection and chronic inflammation. Further, the result showed that the positive rate of HPV 16 progressively increased in the sequential stages from normal epithelia to cancer. These indicated that the malignant progression of oral mucous from normal to dysplasia and cancer might be a result of increased HPV infection proportion from infiltrating inflammation cells. This is consistent with the report of Jin et al[40], who found that TNF-α SNPs may individually or, more likely, jointly affect individual susceptibility to HPV16-associated oral squamous cell carcinoma, particularly OPSCC with never smokers using 325 oral squamous cell carcinoma cases and 176 OPSCC patients. This indicated that chronic inflammation was a biologically important risk factor for HPV-related oropharyngeal carcinogenesis. However, Schäfer et al [41] reported that IL-6 and IL-8 as well as the potential HPV receptor ITGA6 were significantly elevated while IL12A was down-regulated in the tumor tissues. However, none of these genes were expressed in a virus-dependent manner, indicating that HPV infection and inflammation seem to play only a minor role in oesophageal cancer. The difference might be caused by the different geographical and ethnic patients. Much should be done in the future.

In addition, we also detected that there was the higher level of MDSCs in tissues with different degrees of dysplasia compared to normal mucous, as well as the higher level of MDSCs in HPV-positive OPSCC compared to normal mucous, suggesting that the aggregation of MDSCs facilitated HPV-positive oropharyngeal carcinogenesis. MDSCs are known to inhibit the process that cell-mediated immunity protects from tumor occurrence and progression [42, 43]. Therefore, it is likely that the MDSCs induced in an inflammatory process suppressed the activation and function of tumor-specific neutrophil and lymphocytes to promote tumorigenesis. This is in consistence with the findings that MDSCs are found in many cancer patients including head and neck cancers, lung carcinomas, or breast cancers [44–47]. Coussens et al[48] demonstrated that chronic inflammation in some of these malignancies, such as oral, esophageal, and lung cancers, might be susceptible to malignant progression. Serafini et al [49] reported that some of chronic inflammation together with infectious agents associated with MDSCs accumulation. Thus, our results suggest that HPV infection in the microenvironment of chronic inflammation may precipitate oropharyngeal carcinogenesis and MDSCs play an important in this process.

**Fig 4:**
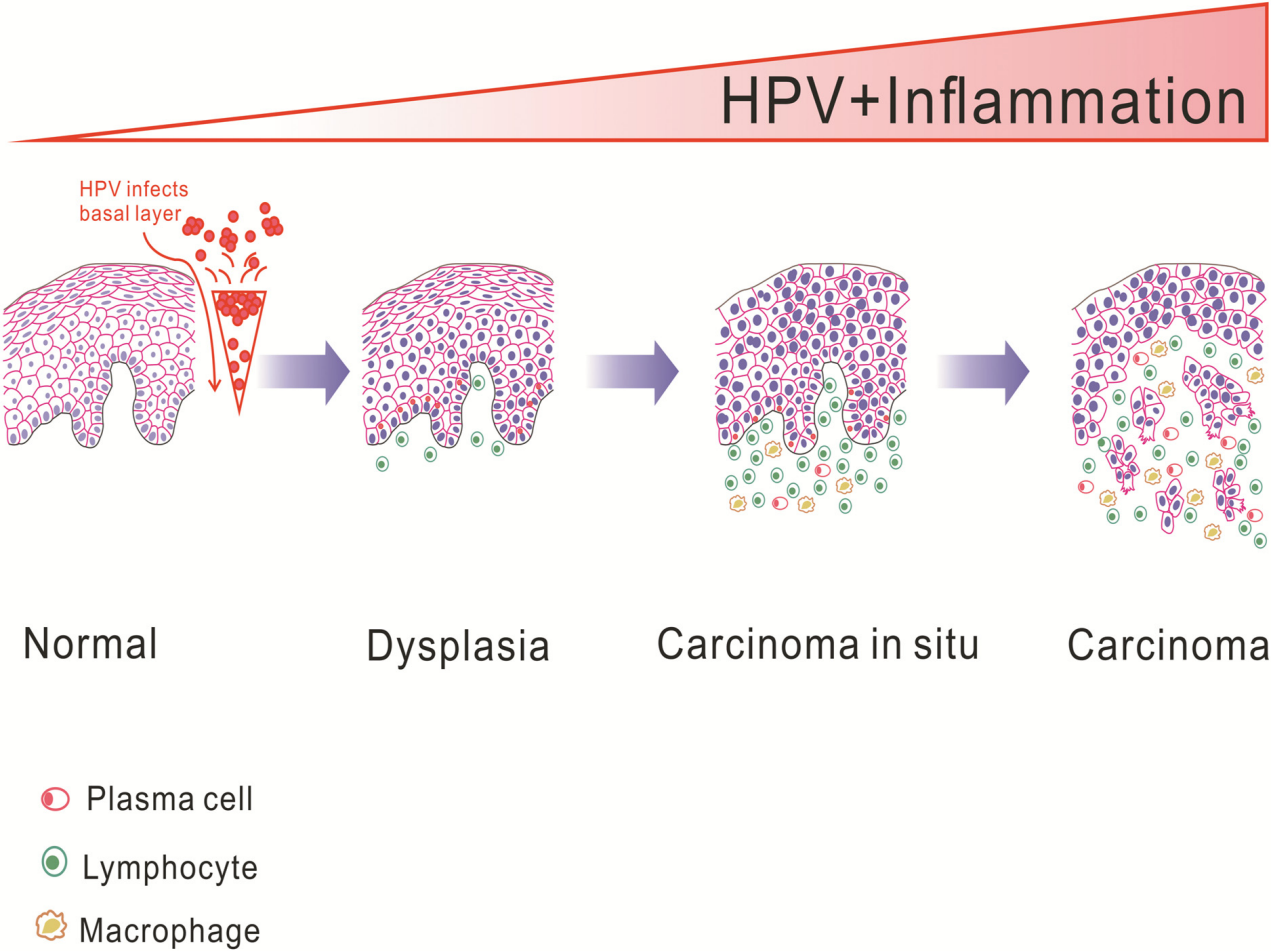
Schematic diagram of persistent chronic inflammation-related HPV infection response contributes to oropharyngeal carcinogenesis.

The observed association between chronic inflammation and HPV infection may be explained through the direct effects of some microorganisms, such as bacteria. Samoff et al [50] have shown that concurrent infection with *Chlamydia trachomatis* could enhance the persistence of cervical HPV infection and the risk of cervical cancer. Yilmaz et al [51]reported that bacteria that infected oral mucous successfully colonized and persisted in the oral mucous. These data indicated that HPV might be triggered in the microenvironment of the chronic inflammation caused by microorganisms. However, Abd Warif et al[52] reported that the expression of HPV 16 E7 in epidermis of the K14E7 mouse could lead to hyperplasia and chronic inflammation. This seems that the chronic inflammation might be caused by HPV, supporting our findings that the higher level of MDSCs presented in dysplastic tissues and HPV-positive OPSCC compared to the normal tissues. Thus, we cannot exclude the dual modulation possibility of the chronic inflammation and HPV infection, and much work should be further done. On the hand, the stimulation of inflammation and immune might pay a critical role in HPV infection. The malignant transformation of oral epithelium would be a consequence of the immune response like MDSCs, macrophage and T-cell activation and cytokine release [53, 54]. The microbial activation of inflammation and immune cells has been shown to contribute to the transformation of malignancy through DNA damage, peroxidation of lipids, or posttranslational modification of proteins [55]. In addition, the relationship between chronic inflammation and HPV infection in oral and oropharyngeal is biologically plausible. The HPV infects exclusively the basal cells of the oral epithelium, replicates in the basal cell and obtains the access through breaks in the oral mucosa (Fig 4). Mucosal damage, microulcerations, and consequent epithelial proliferation mediated by some microorganisms and inflammatory cytokines create an ideal environment for initial HPV infection and its persistence, which leads to increased risk of HPV transmission and oropharyngeal carcinogenesis[56–58].

In conclusion, our present results plus the previous observations support the hypothesis that chronic inflammation and HPV infection contribute to oropharyngeal tumorigenesis and MDSCs play an important role during this process, thereby establishing a favorable microenvironment for tumor progression. Thus, HPV infection may be selected as a promising biomedical marker for indentifying potential oropharyngeal carcinogenesis in individuals with chronic inflammation.
